# Past, Present and Future of the Section “Molecular Toxicology”

**DOI:** 10.3390/ijms24076667

**Published:** 2023-04-03

**Authors:** Guido R. M. M. Haenen

**Affiliations:** Department of Pharmacology and Personalised Medicine, Faculty of Health, Medicine and Life Sciences, Maastricht University, 6200 MD Maastricht, The Netherlands; g.haenen@maastrichtuniversity.nl; Tel.: +31-45-3882109

## 1. Past and Present

**Figure 1:**
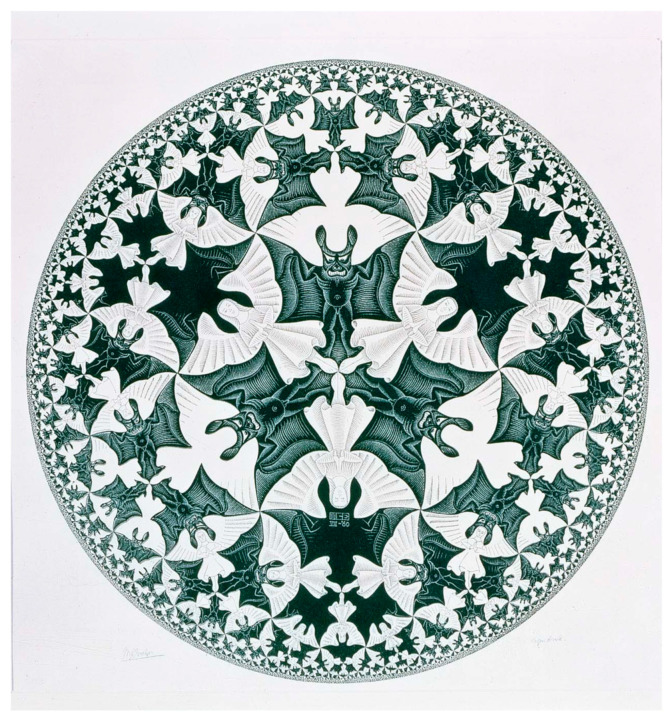
‘Circle limit IV’ by the Dutch master M.C. Escher, which can be considered as a Western visualization of the Eastern Tai Ji symbol. Depending on your perspective, it might on the one hand depict—as described in verse 2 of the Tao Te Ching by Laozi—how opposites create each other. On the other hand, it might also symbolize the unification of two seemingly opposing worlds. This picture is used with the permission of the copyright owner. © 2023 The M.C. Escher Company-The Netherlands. All rights reserved. www.mcescher.com (accessed on 2 March 2023).

‘Forever’ chemicals that unintendedly in the long run pollute the environment, climate change, COVID; life continuously faces all sorts of unforeseen challenges that are an inevitable side product of ‘progress’. Apparently, there is ‘Yin and Yang’ in everything, a concept wonderfully visualized in Figure 1.

Fortunately, humans are resourceful and resilient. Our journal is part of an intricate network through which information flows, allowing scientists to cooperate with each other. Sharing information forms the fundamental basis to ‘understand’ challenges and come up with clues for solutions.

The aim of the section Molecular Toxicology of the International Journal of Molecular Sciences (IJMS) is to rapidly publish contributions that help to elucidate the molecular mechanism of the adverse effects of compounds in cells, animals, populations and the environment, or appropriate models. It is situated at the cutting edge of chemistry and biology and their relation to health. The focus is on the molecule and its interaction with biomolecules, as this will result in the actual biological effect. Manuscripts on molecular methods aimed at preventing toxicity or enhancing our understanding of health risk assessment are also welcome.

The Journal has evolved to a full open access medium, providing free and unlimited access for everyone of manuscripts as well as files of raw data. It performs a transparent and efficient peer-review quality check of submitted manuscripts that also includes a compliance check of ethical guidelines, assures long-term archiving, and is committed to ethical publishing. Moreover, it is indexed in the major scholarly databases to assure high visibility of the presented research.

Statistical analysis shows an increase in parameters used to measure the quality of the Section, for example, manuscripts published in 2022 have already reached 328,856 views and 204,010 downloads, 3.4 times more than was achieved in 2018. In the same period the number of published manuscripts rose from 282 to 641. The Impact Factor rose to 6.2 in 2022.

In 2022, 24 Special Issues were published thanks to the initiatives of our editors. In 2022, we warmly welcomed 11 distinguished scientists who joined us as new Editorial Board Members, namely Dr. Rosaria Scudiero, Dr. Soo-Jin Choi, Dr. Alessandro Di Cerbo, Dr. Anca Oana Docea, Dr. Guido Viel, Dr. Qiu-Gang Ma, Dr. Xi Lin, Dr. Zdenek Dvorak, Dr. Pavel Rossner, Dr. Lijun Wu and Dr. Eric Blomme.

## 2. Future

When we have look to the future, research in molecular toxicology will undoubtedly more focus on in silico computer models and studies with cultured cell systems such as organs on a chip, than on animal testing. One of the strong indicators of this is that before last Christmas, the Government of the United States of America ended the requirement that all new drugs must be tested in at least two animal species before tests in people are allowed [1]. The challenge is to accurately mimic what happens in the in vitro systems to what is going to happen when you administer the drug to a patient, especially with regard to idiosyncratic toxic responses. Elucidating the molecular mechanisms will provide the fundament for this.

Another trend is the ‘safe-by-design’ strategy. Previously the hazard of a new chemical or product was addressed in a relatively late stage of its research and development process, often when the product is already close to market. Looking from all relevant perspectives already at the start of the life cycle of a product, opens the path to proactively ‘design out’ potential hazards. The ‘safe-by-design’ strategy is especially relevant when an innovation is associated with large uncertainties in its biochemical, societal, and ecological consequences.

Terra incognita is the use of artificial intelligence and machine learning technologies for elucidating molecular mechanisms. It might have a lot of potential, but so far it hasn’t delivered. Recent examples of the use of artificial intelligence indicate that the fundament always has to be the use of plain common sense and understanding of what you are doing (comparable to the use of other scientific tools [2]).

**Figure 2:**
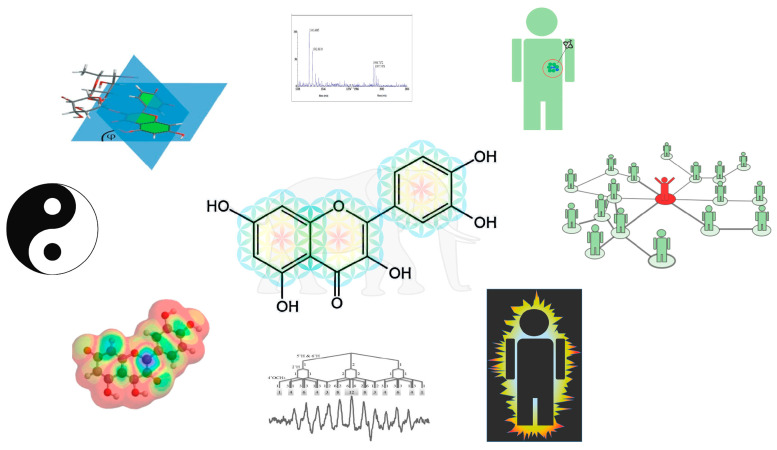
By combining the results obtained with different techniques and from different perspectives, a more complete picture on the effect of compounds can be obtained. Like in the metaphor, you than see that it is not a tree, a wall, a snake, or a rope; it is an elephant. And when you look even closer you might see how the compound is connected to life. Figure adapted from [4].

A path we should remember to find answers is to ‘look back to the future’ to (re)discover strategies provided by for example traditional medicine. There, a more intuitive ritual is practiced, in which the solutions that have slowly evolved over centuries are more soft, and are in harmony with nature. The challenge is to elucidate the underlying mechanism. To come to a solution, you need to look beyond the viewpoint you have; you have to try to combine seemingly opposite views, try to harmonize ‘East and West’ [3] and integrate perspectives, to get a more accurate view (Figure 2) [4]. Egos need to be overcome to find ‘perfect imperfection’.

One of the challenges will be how to keep track of new knowledge with the exponential increase in the number of scientific publications. In my opinion, journals should consider working together, causing new networks to arise such as the interactive ‘WikiPathways’ platform [5], an open community resource dedicated to collecting all interactions among molecules in a cell, and the AOP-Wiki [6] of the adverse outcome pathway (AOP) framework. Within these networks, information is gathered, integrated, and updated by scientists, and discussion points are raised and debated.

I would like to end this farewell address as Editor in Chief to our section with one of the most mysterious interactions I have encountered during my professional career. During and after a war (probably the biggest mistake that can be made), the shortfall of males caused by war appears to be instantaneously ‘corrected’ by an increased percentage of boys among newborns [7,8,9]. Despite the enigmatic nature of the primal force involved, its universal message is crystal clear: ‘Make love, not war’. There is always a ‘third way’ [10]. You, I, we all depend on each other, and—although we are mortal—originate from stardust [11].
